# Quantitative nucleolar proteomics reveals nuclear re-organization during stress- induced senescence in mouse fibroblast

**DOI:** 10.1186/1471-2121-12-33

**Published:** 2011-08-11

**Authors:** Bishnupriya kar, Baohua Liu, Zhongjun Zhou, Yun W Lam

**Affiliations:** 1 Department of Biology and Chemistry, City University of Hong Kong, 88 Tat Chee Avenue, Hong Kong; 2 Department of Biochemistry, The University of Hong Kong, 21 Sassoon Road, Hong Kong

## Abstract

**Background:**

Nucleolus is the most prominent mammalian organelle within the nucleus which is also the site for ribosomal biogenesis. There have been many reports indicating the involvement of nucleolus in the process of aging. Several proteins related to aging have been shown to localize in the nucleolus, which suggests the role of this organelle in senescence.

**Results:**

In this study, we used quantitative mass spectrometry to map the flux of proteins into and out of the nucleolus during the induction of senescence in cultured mammalian cells. Changes in the abundance of 344 nucleolar proteins in sodium butyrate-induced senescence in NIH3T3 cells were studied by SILAC (stable isotope labeling by amino acids in cell culture)-based mass spectrometry. Biochemically, we have validated the proteomic results and confirmed that B23 (nucleophosmin) protein was down-regulated, while poly (ADP-ribose) polymerase (PARP) and nuclear DNA helicase II (NDH II/DHX9/RHA) were up-regulated in the nucleolus upon treatment with sodium butyrate. Accumulation of chromatin in the nucleolus was also observed, by both proteomics and microscopy, in sodium butyrate-treated cells. Similar observations were found in other models of senescence, namely, in mitoxantrone- (MTX) treated cells and primary fibroblasts from the Lamin A knockout mice.

**Conclusion:**

Our data indicate an extensive nuclear organization during senescence and suggest that the redistribution of B23 protein and chromatin can be used as an important marker for senescence.

## Background

Cellular senescence is an irreversible growth-arrest programme that limits cell proliferation in an organism. This process may contribute to physiological aging, a complex phenomenon that involves the interplay of multiple genetic and environmental factors, resulting in the diminishing capacity of tissues to respond to stress and injury [1,2]. Many experimental models have been established for studying this fundamental process. Replicative senescence is one of the most commonly used model systems. Some of the morphological and molecular events in senescence can be reproduced by the repeated passage of mammalian cells in tissue culture, possibly due to telomere deprotection. Senescence can be accelerated by subjecting cultured cells to chemical or genotoxic stress, such as sodium butyrate [3,4], MTX [5], irradiation [6,7] or by overexpressing the oncogenic ras gene [8]. In addition, the contributions of specific genes to the regulation of senescence have been studied by using cell lines derived from patients of accelerated aging diseases, such as Werner's disease [9]. Finally, transgenic animals have been created as models for accelerated aging diseases [10,11]. For example, Lamin A knockout mice manifest symptoms of pre-mature aging diseases. Another line of transgenic mice that lacked Zmpste24, an enzyme that aids in the maturation of pre-lamin A to Lamin A, also demonstrate accelerated aging phenotypes [12,13].

The association of mutations in lamins and lamin-related proteins with accelerated aging phenotypes in humans suggests the possible role of nuclear shape and organization in the regulation of senescence. It is known that the nuclear architecture changes during the natural aging of wild-type Caenorhabditis elegans [14]. Major changes in chromatin organization, with the formation of senescence-associated heterochromatin foci (SAHF), have also been observed in cultured human fibroblasts when senescence was induced by overexpressing the ras oncogene [15]. Apart from the nuclear envelope and chromatin, the most common cellular orgenelle associated with aging is the nucleolus. Many of the proteins mutated in human premature aging syndromes [9] are localized in the nucleolus at least during part of the cell cycle or under some other conditions. For example, the wild-type Werner syndrome protein is partially localized in the nucleoli, where it may be involved in rDNA transcription [16]. Similarly, the Cockayne syndrome B (CSB) protein is also nucleolar [17]. The BLM protein, mutated in the Bloom syndrome, is present in the nucleolus during the S phase [18], while the Rothmund-Thomson disease protein RecQL4 accumulates in the nucleolus during oxidative stress [19]. In addition, dyskerin, a protein mutated in the premature aging disease, dyskeratosis congenital, is associated with snoRNAs [20] and telomerase RNA, are also localized in the nucleoli and Cajal bodies [21]. The intranuclear localization of these proteins hint at the possible role of the nucleolus in aging in response to cumulative stress sustained by cells over time, or to the maintenance of the proliferative capacity of stem cells essential for tissue repair.

A more direct demonstration of the role of nucleoli in aging comes from observations in which some mutations in budding yeast can lead to an extension in life span [22]. For example, cells with mutations in the SIR4 gene can live for 50% longer. SIR4 encodes a protein that exists in a complex with Sir2p and Sir3p, which is linked with transcriptional silencing. Remarkably, Sir3p is localized at the telomeres and HM loci in young yeast cells, but relocate to the nucleolus in old cells. Furthermore, life-extending mutations of SIR4 can also cause the SIR2/3/4p complex to accumulate in the nucleolus. These findings show that, at least in yeast, the composition of nucleoli changes as the cell ages, and that these changes are functionally linked to the control of lifespan. The redistribution of Sir protein from telomeres to nucleoli as yeast ages is echoed by the detection of telomeric components in mammalian nucleoli. Tomlinson et al. [23] examined the intracellular distribution of telomerase RNA and telomerase reverse transcriptase in HeLa cells, and reported their localization in nucleoli and Cajal bodies during the S phase, which implicates that both these structures are involved in the biogenesis and trafficking of telomerase. Telomerase can also interact with nucleolin [24], an abundant multi-functional nucleolar protein. In mammalian cells, telomere shortening initiates senescence by activating p53, the central regulator of cellular stress responses, and thereby signalling to the cell cycle checkpoint pathways, which are also activated by DNA damage [25]. The nucleolus once again plays a crucial role in regulating the activity and stability of p53 [26]. The nucleolar protein ARF regulates the ubiquitination and degradation of p53. Other p53 interacting proteins, such as nucleostemin [27], and ING1b [28] are also found in the nucleoli, which suggests that nucleolar targeting of p53-regulating proteins is a common mechanism for the activation of p53 and hence, all p53-related pathways. Notably, nucleostemin is a nucleolar GTP-binding protein highly enriched in the stem cells and cancer cells, but absent in differentiated cells [27]. Manipulation of nucleostemin expression levels leads to changes in senescent phenotypes of mouse cells [27].

The nucleolus is the largest organelle in a mammalian nucleus and the site of ribosomal subunit production. Recent studies have revealed that nucleolus also plays a significant role in other cellular functions. New techniques for studying the nucleolus are starting to provide insights into these non-conventional functions of this organelle. The human nucleolus has been the subject of some of the largest organellar proteomic efforts [29]. Almost 700 proteins have been characterized, and out of which at present time about 30% are uncharacterized genes. SILAC-based quantitative mass spectrometry has been successfully applied to study the effect of transcription inhibition on the protein composition of the nucleolus [30]. More recently, this technique has been used to study the effect of viral infections on HeLa cell nucleolar proteome [31]. In this study, we used this high throughput quantitative mass spectrometry-based approach to map global changes in nucleolar proteome in stress induced senescent cells and provide for the first time, a biochemical snapshot of this organelle in senescent mouse fibroblast cells.

## Methods

### Cell culture

The mouse fibroblast cell line, NIH3T3 cells and primary mouse fibroblast cells were grown in Dulbecco's Modified Eagle Medium (DMEM) (GIBCO, Invitrogen, USA) supplemented with 10% FBS with Anti-anti (100 units/ml) (GIBCO, Invitrogen, USA), maintained at 37°C, 95% humidity, and 5% carbon dioxide.

### Reagents and antibodies

Sodium butyrate and MTX were purchased from Sigma-Aldrich. A 2 M stock solution of sodium butyrate was made in distilled water and stored at 4°C. A 2 mM stock solution of MTX was made in ethanol and stored at 4°C. Antibodies for fibrillarin (H-140), PARP (H-300) and NDHII (H-300) were purchased from Santa Cruz Biotechnology. The antibodies for B23 and actin were purchased from Sigma-Aldrich. A fluorescence conjugated secondary antibody (anti-mouse Ig -TRITC) was purchased from Jackson Immunologicals. Anti-mouse-HRP and Anti-rabbit-HRP were purchased from GE Healthcare. A Novex colloidal blue staining kit was purchased from Invitrogen for staining protein bands with coomasie blue on sodium dodecyl sulfate polyacrylamide gel electrophoresis (SDS-PAGE) gel.

### Senescence associated (SA) β-galactosidase staining

A senescence β-galactosidase staining kit was purchased from Cell Signaling Technology. NIH3T3 cells were seeded on a six-well tissue culture dish, and the next day, treated with 5 mM sodium butyrate. Following 4 days of treatment with sodium butyrate, the cells were stained with β-gal according to the manufacturer's protocol. The cells were then monitored for blue coloration under a microscope.

### Immunostaining and microscopy

For indirect immunofluorescence, the cells were cultured on coverslips that were sterilized by dipping into 100% ethanol followed by flaming. The cells were fixed with 4% paraformaldehyde in PBS for 30 min at room temperature and then permeabilized with 0.2% Triton X-100 in PBS for 5 min at room temperature, washed in PBS, and blocked with 50 mM glycine in PBS for 10 min. The coverslips were washed and incubated in either a wash buffer (control) or primary antibody at a dilution of 1:100-1:400 for 1 h at room temperature and then washed three times in PBS and stained with a fluorescence-conjugated secondary antibody at a concentration of 1:100 (Jackson Immunologicals) for 1 h at room temperature and counter-stained with Hoechst 33258 (Sigma). The coverslips were mounted on a glass slide in a fluorescent mounting medium (Dako). Images were captured with a confocal microscope (Leica TCS SPE).

### 5-Fluorouridine incorporation

In situ assay of RNA synthesis by 5-Fluorourdine (5-FU) incorporation was performed as described [32]. Briefly, 5-FU (Sigma-Aldrich) was added to the culture medium to a final concentration of 1 mM. After an incubation in normal tissue culture conditions for 10 minutes, the cells were washed in PBS and fixed in 1% paraformaldehyde (room temperature, 5 min) and then permeabilized with 0.5% Triton X-100 in PBS for 5 min at room temperature. The incorporated 5-FU was detected by anti-BrU antibody (1:500, Sigma B2531) and fluorescence-conjugated secondary antibody as described above.

### Immunoblotting

The protein samples were separated with NuPAGE 12% Bis-Tris gels (Invitrogen). Western blotting was performed following a protein transfer from NuPAGE gel to polyvinylidene difluoride (PVDF) membranes (Millipore). Blots were blocked in a 5% nonfat dry milk solution made up in PBS. The blots were subsequently incubated with the appropriate primary antisera for 1 h at room temperature. The membranes were washed three times for 10 min each time in a PBS solution that contained 0.05% Tween-20 solution. Subsequently, secondary antibodies conjugated to a horseradish peroxidase (GE Healthcare) were added and incubated at room temperature for 1 h. The blots were developed with an enhanced chemiluminescence kit (GE Healthcare).

### FACS analysis

Cells (5 × 10 ^5^) were harvested by trypsinization, washed in PBS (200 g, 2 mins) and fixed in 70% ice-cold ethanol for 1 h at 4°C. Following fixation, the cells were washed in PBS twice and re-suspended in 500 μL of staining solution (10 μg/mL RNase A and 50 μg/mL propidium iodide in PBS). The tubes were incubated for 30 min at 4°C in the dark. The DNA content was analyzed by a flow cytometry (BD Calibur) and the percentage of cells in different phases of the cell cycle was assessed by using ModFit V 3.0 software.

### Nucleolar isolation

Nucleolar isolation was performed according to the published protocol with the following modifications for NIH3T3 cells http://www.lamondlab.com/f7nucleolarprotocol.htm. The NIH3T3 cells were dounce homogenized 50 times which is enough to break open the plasma membrane without disrupting the nucleus. We sonicated the isolated nuclei for 12 × 10 sec at amplitude of 30% (Cole-Parmer Instruments) to break open the nuclear membrane without disrupting the nucleolus.

### SILAC labeling and mass spectrometry

For SILAC labeling, prior to nucleolar isolation, all cells were metabolically labeled by growing five rounds of cell divisions in a SILAC DMEM medium supplemented with 10% dialysed fetal calf serum (Biowest) to ensure all the cellular proteins were labeled to saturation. All isotopically labeled amino acids were purchased from Cambridge Isotope Lab. Untreated control cells were labeled with DMEM that contained L-13C6-arginine (R6) and L-4,4,5,5-D4-lysine (K4), whereas sodium butyrate-treated cells were cultured in DMEM that contained L-arginine (R0) and L-lysine (K0).

Protein amounts of nucleolar lysates were estimated by a Bradford assay (Pierce). Nucleolar protein fractions from control and treated cells were mixed at a 1:1 protein amount. The combined protein sample was separated by electrophoresis as above. The gel was then fixed (50% methanol, 10% acetic acid, 40% ddH_2_O), stained with colloidal coomassie blue (Invitrogen) according to the manufacturer's instructions. The gel lane was cut into 15 slices. The gel slices were destained with a destaining solution (Trypsin Profile IGD kit from Sigma) according to the manufacturer's instructions, followed by incubation in 50 mM ammonium bicarbonate that contained proteomic grade Trypsin (Sigma) (50 ng/gel slice) overnight at 37°C. The resulting peptides were extracted from each gel slice, analyzed by LC-MS on an LTQ-Orbitrap mass spectrometer system (ThermoElectron) coupled to a Dionex 3000 nano-LC system (Camberley). The peptide mixture was loaded onto an LC-Packings PepMap C18 column trap column (0.3 × 5 mm) equilibrated in 0.1% TFA in water at 20 μl/min, and washed for 3 min at the same flow rate, and then the trap column was switched in-line with an LC-Packings PepMap C18 column (0.075 × 150 mm) equilibrated in 0.1% formic acid/water. The peptides were separated with a 55-min discontinuous gradient of acetonitrile/0.1% formic acid (2-40% acetonitrile for 40 min) at a flow rate of 300 nl/min and the HPLC was interfaced to the mass spectrometer with an FS360-20-10 picotip (New Objective) fitted to a nanospray 1 interface (ThermoElectron) with a voltage of 1.1 kV applied to the liquid junction.

The Orbitrap was set to analyze the survey scans at 60,000 resolution and the top five ions in each duty cycle selected for MS/MS in the LTQ linear ion trap. The raw files were processed to generate a Mascot generic file by using the program Raw2msm [33] and searched against the UniProt human database by using the Mascot search engine v.2.2 (Matrix Science) run on an in-house server with the following criteria; peptide tolerance = 10 ppm, trypsin as the enzyme and carboxyamidomethylation of cysteine as a fixed modification. Variable modifications were the oxidation of methionine, and appropriate SILAC labels. Proteins with a total Mascot peptide scores below 35 were ignored, based on the decoy reversed database searching.

Quantitation was performed by using the program MS-Quant http://msquant.sourceforge.net, with peptide ratios calculated for each arginine- and/or lysine-containing peptide as the peak area of labeled arginine/lysine divided by the peak area of nonlabeled arginine/lysine for each single-scan mass spectrum. Peptide ratios for all arginine- and lysine-containing peptides sequenced for each protein were averaged. MS-Quant measures the SILAC ratio of each peptide throughout the elution of the ion pairs [34], generating an averaged ratio for the identified peptide. Standard deviations of the averaged protein ratios estimate the variability of measured SILAC ratios for all the detected peptides for each protein. Individual spectra were inspected by using QualBrowser software (XCalibur; ThermoElectron). ProteinCenter (Proxeon Bioinformatics) proteomics data mining and management software was used to eliminate redundancy and compare datasets, and convert protein IDs to gene symbols and perform initial gene ontology characterization.

## Results and Discussion

### Sodium butyrate induces senescence in NIH3T3 cells

**Figure 1:**
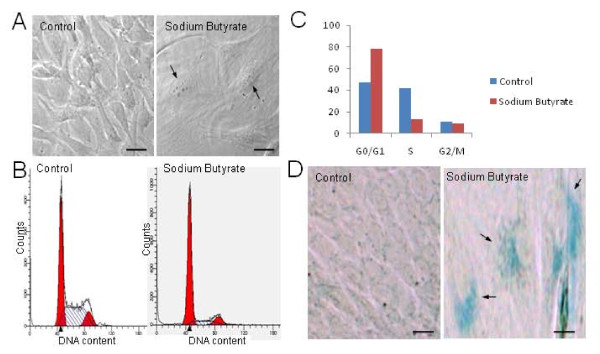
**Induction of senescence in NIH3T3 cells following sodium butyrate treatment (A) NIH3T3 cell was treated with 5 mM of sodium butyrate for 4 days and the morphological changes were monitored under a microscope**. The bar represents 10 μm. (B) A FACS analysis of the control and treated NIH3T3 cells followed by propidium iodide staining to analyze the cell cycle progression. Percentage of untreated or sodium butyrate treated NIH3T3 cells in different phases of the cell cycle analyzed by using ModFit V 3.0 software. (C) A graphical representation of the percentage of cells in different cell cycle phases in control and Sodium Butyrate treated cells as determined by propidium iodide staining and followed by FACS analysis. (D) SA-β-gal staining of the control and sodium butyrate treated NIH3T3 cells following 4 days of treatment. Blue coloration in cells indicates senescence.

We used sodium butyrate treatment of NIH3T3 cells as a model system to study the effect of senescence on nucleolar proteome in mammalian cells [4]. Following treatment with 5 mM sodium butyrate for 4 days, NIH3T3 cells adopted a flattened morphology with spread-out cytoplasms, large nuclei (arrows, Figure 1A) and in many cases, the cells were multinucleated. The treated cells grew at a slower rate than the control cells, but with little or no discernible cell death (data not shown). A flow cytometry analysis showed that the treated cells are blocked in the G0/G1 phase of the cell cycle with a reduced S-phase population (Figures 1B, C). To confirm that the phenotypes we observed were results of chemically induced senescence, but not pharmacological cell cycle blockage, we performed a senescence associated (SA)-β-gal assay on the sodium butyrate-treated and mock-treated cells. SA-β-gal assay measures the activity of β- galactosidase, a widely used biomarker for senescent cells [35]. As shown in Figure 1D, the NIH3T3 cells show blue coloration (arrows, Figure 1D) after 4 days of sodium butyrate treatment, whereas control cells are unstained. Taken together, sodium butyrate induces senescence in NIH3T3 cells.

### Isolation of nucleolus from normal and senescent NIH3T3 cells

**Figure 2:**
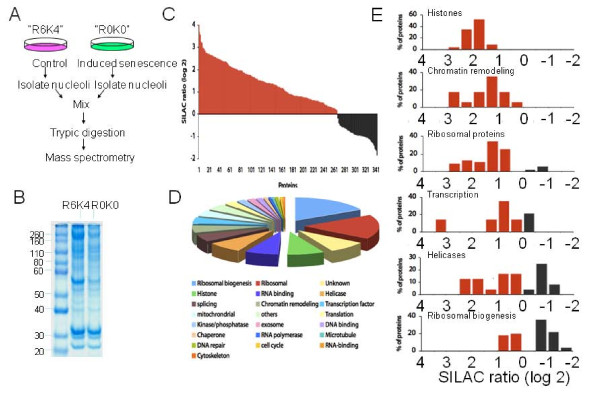
**Nucleolar proteome following senescence induced by sodium butyrate treatment in NIH3T3 cells (A) SILAC labeling of NIH3T3 cells to determine nucleolar protein dynamics in sodium butyrate induced senescent cells**. The workflow shows SILAC labeling of cells in a heavy (R6K4) media and a light (R0K0) media for 5 days followed by treatment of cells with vehicle or sodium butyrate. The cells grown in the lighter media (R0K0) were treated with sodium butyrate for 4 days. Subsequently, the nucleolus from both control and sodium butyrate treated cells were isolated. (B) Coomasie staining of the SDS PAGE gels of control and sodium butyrate treated nucleolar lysates from cells grown in SILAC media. The protein nucleolar lysates were quantitated using Bradford-Lowry reagent and 50 ug of total nucleolar lysate were run on a SDS-PAGE. Note the changes in the protein composition of control and treated nucleolar lysate. (C) Dynamic regulation of nucleolar proteins identified from quantitative proteomics by using SILAC based LC MS/MS following senescence induced with sodium butyrate in NIH3T3 cells. SILAC ratio was defined by the log2 of the averaged R0K0/R6K4 ratios of the peptides identified for each protein. (D) Functional classification of 344 proteins identified from SILAC based mass spectrometry in sodium butyrate induced senescent NIH3T3 cell nucleoli. (E) Analysis of the fold change of the proteins in the nucleolus following sodium butyrate induced senescence based on their cellular function.

To study the changes in the nucleolar protein composition following senescence induction in NIH3T3 cells, we performed SILAC-based quantitative proteomics on nucleoli isolated from mock and sodium butyrate treated cells. In this approach, a population of NIH3T3 cells were cultured in a medium that contained heavy isotope-labeled arginine and lysine (R6K4, see experimental procedure), and another population in the same medium, except that the arginine and lysine were not isotopically labeled (R0K0). To ensure complete labeling, the cells were first cultured in these media for 3 days, and then for the next four days, they were either mock treated (R6K4) or sodium butyrate treated (R0K0) while still in their respective labeled media (Figure 2A). There was no observable effect of the use of SILAC media on cell growth and responses to sodium butyrate (data not shown). Following the treatment, nucleoli were isolated from four 15 cm dishes of treated cells and the same number of untreated control by using an established protocol. The nuclei were first isolated from the cells, and the nucleoli were released from the nuclei by sonication and then purified from the nucleoplasmic fraction by a sucrose cushion. A microscopy analysis of the isolated nuclei and nucleoli (Additional file 1, A) demonstrated the intactness and purity of these fractions. A Western blotting analysis of the cytosolic, nucleoplasmic and the nucleolar lysates with antibodies for fibrillarin (nucleolar marker), Lamin A/C (nucleoplasmic marker) and actin (cytoplasmic marker) confirmed the purity of that of the respective subcellular fractions (Additional file 1, B). Equal amounts of proteins from nucleoli isolated from control and butyrate-treated cells were mixed, separated by SDS-PAGE, and in-gel digested by trypsin (Figure 2B). The resulting peptides were analyzed by nano-ESI-LC-MS/MS.

### The nucleolar proteome during senescence

We have identified and quantified a relative abundance of 344 proteins from a proteomic comparison of the senescent and control nucleoli (Figures 2C and 2D, Additional file, 2). The proteomic analysis indicated that the detected proteins in mouse NIH3T3 nucleoli cover a similar functional spectrum as the human nucleolar proteomes previously reported [36,37]. This confirms the strong conservation of the compositions and functions of the nucleolus [38]. The response of the nucleolar proteome to chemically induced senescence is highly distinct from that of other perturbations [30,31]. With some notable exceptions, most nucleolar proteins appear to be depleted from the nucleolus as a result of transcriptional inhibition by actinomycin D treatment [30]. In sodium butyrate treated NIH3T3 cells, however, there is an overall accumulation of proteins in the nucleolus, with only a small subset of proteins found to be down-regulated following the treatment (Figure 2C). This observation demonstrates the remarkable plasticity of the nucleolus, which responds differently to distinct metabolic conditions. For example, ribosomal proteins are shown to accumulate in the nucleolus of sodium butyrate treated cells (Figure 2E), but depleted from the nucleolus in actinomycin D treated cells whereas in adenoviral infected cells, the abundance of ribosomal proteins in the nucleolus is largely unaffected [31]. Interestingly, many proteins related to ribosomal biogenesis, such as Pop1, RrP15, Rrp12, BAP28, are depleted from the nucleolus after sodium butyrate treatment. The opposite behaviours of ribosomal proteins and ribosomal biogenesis factors suggest a possible shift in the kinetics of ribosome assembly in senescent cells. Histones and chromatin remodeling factors, such as CHD5, RBBP4, and CHD4, also dramatically accumulate in the nucleolus upon treatment with sodium butyrate, which implies a major change of chromatin organization in the nucleolus during senescence (Figure 2E). Taken together, our quantitative proteomic analysis offers a unique overview of the biochemical impact of chemically induced senescence on the nucleolus.

### Biochemical Validation of the Proteomic results from Mass spectrometry

**Figure 3:**
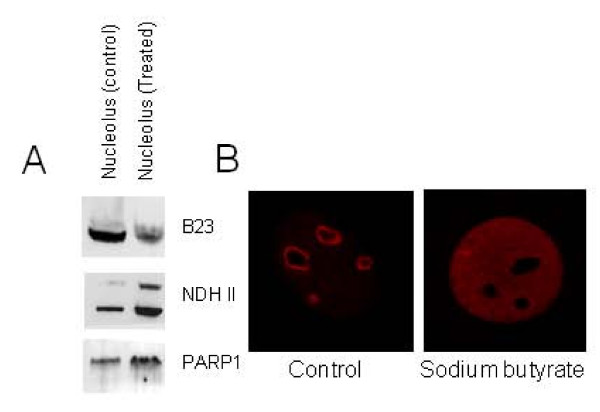
**Validation of proteomic result (A) Western blotting analysis used to validate the mass spectrometric result**. NIH3T3 cells were vehicle or sodium butyrate treated for 4 days followed by isolation of nucleoli. The protein concentration was ascertained and then 10 ug of total protein was run on an SDS-PAGE to compare the levels of B23, NDH II and PARP1 proteins. Western blotting was performed with anti-B23, anti-NDH II and anti-PARP1 to validate the levels of these proteins following sodium butyrate induced senescence. (B) Indirect Immunofluorescence showing B23 localization in control and sodium butyrate treated (4 days) NIH3T3 cells.

We selected three proteins identified by proteomic analysis out of 344 proteins that were shown to change in abundance in nucleoli in sodium butyrate treated cells, and validated the SILAC results by traditional techniques. Following sodium butyrate induced senescence, the nucleolar amount of B23 was down-regulated with a ratio of 0.59, while NDHII (DHX9/RHA) and PARP1 were up-regulated with a ratio of 11.9 and 4.29, respectively (Additional file 2). The amount of B23, NDH II and PARP1 in the nucleolar lyaste of sodium butyrate treated cells was compared to control cells by Western blotting as shown in Figure 3A. We found that the B23 protein level is indeed down-regulated while the protein levels of PARP and NDH II are up-regulated following the sodium butyrate treatment. To further characterize the decrease of B23 in the nucleolus of senescent NIH3T3 cells, we observed the subcellular distribution of B23 in treated and control cells by immunofluoresence. Consistent with the MS and Western blotting results, the nucleolar staining by anti-B23 in sodium butyrate treated NIH3T3 cells is clearly weaker than that in untreated cells (Figure 3B). Interestingly, there is a corresponding increase of B23 labeling in the nucleoplasm of the sodium butyrate cells, which suggests that the observed decrease in the abundance of nucleolar B23 may reflect a re-distribution of this protein from the nucleolus to the nucleoplasm.

### Reorganization of the nucleolus after sodium butyrate treatmet

**Figure 4:**
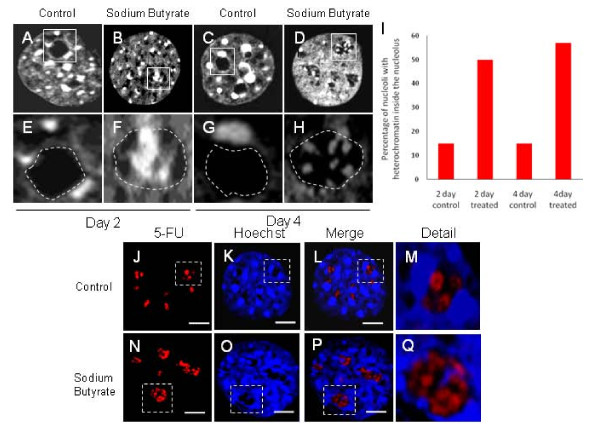
**Analysis of chromatin organization in sodium butyrate induced senescent NH3T3 cells**. (A-D) NIH3T3 cells were treated with sodium butyrate for 2 or 4 days and then stained with Hoechst 33258 for visualizing chromatin. (E-H) is a magnified view of the field in boxes in A-D respectively. (I) Quantitative analysis of the nucleoli in sodium butyrate treated cells with chromatin re-organized from outside to inside the nucleolus. About 200 nucleoli were counted in the treated and control cells and the number expressed as a percentage of the nucleoli in which chromatin was found to be inside the nucleoli. (J-Q) Assessment of in situ rRNA transcriptional sites in the 4 day sodium butyrate treated senescent NIH3T3 cells by 5-fluorouridine labeling followed by staining with BrdU antibody (Red). (J-M) depicts control cells while (N-Q) depicts sodium butyrate treated cells. The detailed magnified view of the areas in broken boxes is shown in M and Q.

From our proteomic analysis, we observed that there was an upregulation in the proteins related to chromatin remodeling following induction of senescence in NIH3T3 cells with Sodium butyrate. To further study this effect, we stained the control and Sodium butyrate treated cells with DNA dye Hoechst 33342 and observed the nuclei with high magnification under confocal microscope. When the butyrate-treated NIH3T3 cells were stained with DNA dye Hoechst 33342, compared to control cells (Figures 4A, E, C 4G), we observed the presence of chromatin within the nucleoli of many treated cells (Figures 4B, F, D, H). This probably reflects the increase in abundance of histones and other chromatin-related proteins as detected by MS. In untreated and mock-treated NIH3T3 cells, clusters of chromatin centers (visualized by Hoechst staining) could be seen in the interphase nucleus, frequently associated with the nucleolar periphery, which are commonly observed in mouse cells. The core of the nucleoli is generally free of chromatin, an observation consistent with early electron microscopy studies [39]. Within 2 days of sodium butyrate treatment, however, Hoechst-labeled chromatin is found on the inside of some of the nucleoli of the treated NIH3T3 cells (Figure 4F). To quantitate the number of nucleoli that display intranucleolar chromatin, we counted 200 nucleoli in both controls, and 2 or 4 day sodium butyrate treated cells. We observed that there is about a 40% increase in the proportion of cells displaying nucleolar chromatin following 2 or 4 days of treatment (Figure 4I).

### Sodium butyrate-induced nucleolar organization does not affect nucleolar RNA synthesis

Since heterochromatin is often associated with transcriptional silencing, we asked if the sodium-butyrate-induced accumulation of chromatin in the nucleolus is a reflection of an inhibition of nucleolar transcription. Sodium butyrate-treated and mock-treated NIH3T3 cells were pulsed with 5-FU and then immuno-cytochemically labeled for transcription sites (Figures 4J-Q). As shown in Figure 4N, there was no detectable difference in the amount of 5-FU incorporation in the nucleolus after a sodium butyrate treatment, which indicates that sodium butyrate does not affect the rate of RNA synthesis in the nucleolus. The appearance of chromatin inside the nucleolus and the re-distribution of B23 suggest a general re-organization of the nuclear structure as a result of the sodium butyrate treatment.

### Validation of results by using other senescence model systems

The c-Jun NH^2^-terminal kinase (JNK) is a mitogen-activated protein kinase that has been implicated to be activated in response to stress in many cell lines, but increasing evidence also suggests that JNK plays a role in cell proliferation and survival [40] too. It has been also demonstrated that in UV irradiated cells following JNK activation, c-Jun translocates B23 out of the nucleolus [41]. Since sodium butyrate induces an arrest of NIH3T3 cells at G1, it is important to verify whether the observed redistribution of B23 in the nucleolus in sodium butyrate treated cells is a senescence-specific event and not a collateral result of stress. To this end, we examined the nucleolar abundance of B23 in serum-starved NIH3T3 cells for 24 hours. A FACS analysis showed that over 98% of the cells are blocked in the G1 phase following serum starvation (Additional files 3, A, B). To investigate the level of B23 protein, we isolated nucleoli from control and serum starved cells, and the protein amount was quantitated by using the Bradford method. Equal amounts of nucleolar protein were run on an SDS-PAGE. A Western blotting analysis with an anti-B23 antibody showed that there is no observable alteration in the level of B23 protein in the serum starved cells compared to the control (Additional file 3, C). This result demonstrates that the down-regulation of B23 protein levels in the nucleolus is not a general phenomenon of the G1 cell cycle block, but a sodium butyrate specific effect.

**Figure 5:**
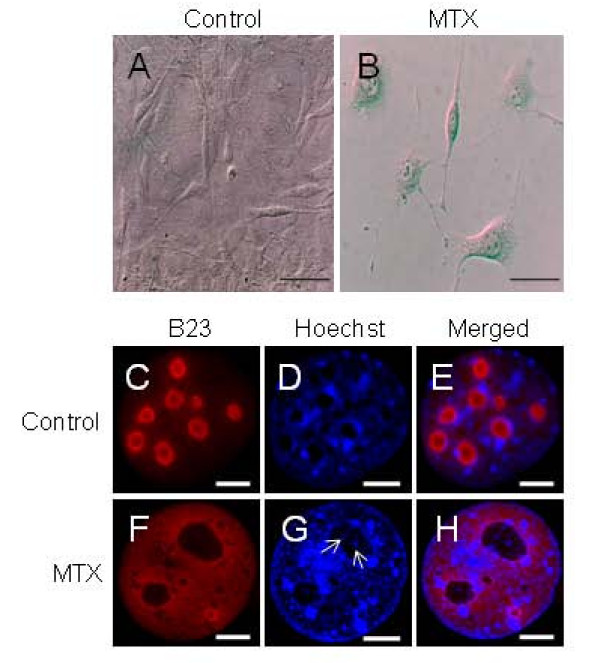
**Effect of MTX treatment on B23 localization in NIH3T3 cells (A, B) Morphological changes and SA-β-gal staining of NIH3T3 cells when treated with 1 uM MTX for 9 days**. The blue coloration is an indication of senescence. (C-H) B23 protein staining in MTX treated NIH3T3 cells. The cells were also counter stained with Hoechst 33258 to stain DNA. The arrows in G point to chromatin foci inside the nucleolus of MTX treated cells. The bars represent 10 μm.

Mitoxantrone (MTX) treated cells have been shown to undergo genotoxic stress-induced senescence in fibroblast cell lines by many laboratories [42]. Since the cellular target for MTX is different from sodium butyrate, we chose to confirm our finding through MTX treatment with a concentration that has been known to induce senescence in cells. Nine days after the MTX treatment, the NIH3T3 cells demonstrated senescent morphology, with larger and flattened cells, and bigger nuclei which were positive in the SA-β-gal assay (Figures 5A, B). We observed little toxicity, but also a slower proliferation in the MTX treated NIH3T3 cells. Upon a FACS analysis of the propidium iodide stained MTX treated cells, we found 80% of the treated cells to be blocked in the G2/M phase after 2 days and following 9 days of treatment, a significant percentage (45%) of the cells are still blocked in the G2 phase (Additional file 4, A, B), in concurrence with the literature [43]. Following 9 days of MTX treatment of NIH3T3 cells, we fractionated the cells and checked for purity of the preparation by Western blotting using B23 (nucleolus), lamin A/C (nucleus) and Actin (cytoplasm) as markers (Additional file 4, C). We found that upon MTX treatment, like sodium butyrate, leads to a redistribution of B23 to the nucleoplasm (Figure 5C-H). The effect of MTX treatment on the level of nucleolar B23 protein is even more pronounced than sodium butyrate. Furthermore, a similar appearance of chromatin in the nucleolus was also observed in MTX treated cells (arrow, Figure 5G), which confirms that the observed re-organization of the nuclear structure in sodium butyrate treated cells is not a cellular response to particular chemicals, but more likely a general reflection of senescence.

**Figure 6:**
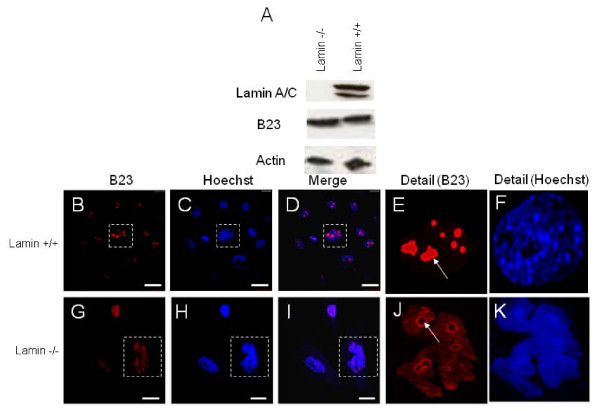
**Comparison of the distribution of B23 protein in primary fibroblast cells from lamin -/- and lamin +/+ mice (A) lamin +/+ and lamin -/- cells were lysed in RIPA buffer and Western blotting performed with antibody for lamin A/C, B23 or actin**. The lysates were quantitated using Bradford-Lowry reagent and then 10 ug of lysate was run on a SDS-PAGE. (B-K) lamin A +/+ and lamin A-/- cells were grown on coverslips and then stained for B23 protein. The cells were also counter stained with Hoechst 33258 to stain DNA. The arrows in (E) and (J) point to the representative nucleolar staining of B23 in Lamin A +/+ and Lamin A -/- cells respectively. The bars represent 10 μm.

To further confirm the observed nuclear re-organization in senescent cells, we utilized an animal model of aging. Mutations in LMNA have been shown to cause a whole range of pre-mature aging diseases, like the Hutchinson-Gilford progeria syndrome (HGPS), Werner's syndrome (WRN) and cardiocutaneous progeria syndrome (CCPS) [11,12,44-46]. Embryonic fibroblasts derived from Lamin A knockout mice are a widely used model system to study senescence [47,48]. To investigate if MEFs derived from the knockout mice display any changes in the B23 protein expression, we performed immunostaining for B23 protein. As expected, although we did not observe any changes in the total B23 protein level in the control and knockout MEFs (Figure 6A), but the distribution of B23 in the two compared cell lines are significantly different. We found that the MEFs from knockout mice display diffused staining in the nucleus for B23 (Figure 6G-K) as opposed to nucleolar staining in the control cells (Figure 6B-F), with the appearance of chromatin in the nucleoli (Figure 6K), which is consistent with our data in cells induced by either sodium butyrate or MTX. Taken together, the redistribution of B23 and heterochromatin was observed in the nucleoli of senescent cells irrespective of the stimuli, and this suggests that it is a senescence specific effect and not due to the agent used to induce senescence.

There have been sporadic reports on the presence of condensed chromatin in the proximity of the nucleolus. For example, in human lymphocytes, it has been observed that some chromatin inclusions reside close to the fibrillar center in the nucleolus [49]. Although dramatic heterochromatin reorganizations, including the formation of SAHF, have been observed in senescent cells [50], but these structures are not reportedly within the nucleolus. Interestingly, we observed that in sodium butyrate treated mouse fibroblast cells, the chromatin re-organizes and relocates inside the nucleolus. Histone H1x exhibits a cell-cycle-dependent change in its nuclear distribution and accumulates in the nucleolus during the G1 phase when treated with sodium butyrate and it was evenly distributed in the nucleus during the S phase and G2 [51]. In our proteomic screening of sodium butyrate treated mouse fibroblast senescent cells, we observed a general accumulation of histones in the nucleolus. We observed the same phenomenon in MTX-treated senescence cells, which is a G2 cell cycle block. We similarly also observed a chromatin relocation to the nucleolus in the primary *Lmna*-/- mouse cells which is a well known mouse model system for studying pre-mature aging. So, we believe that it is not an effect of a cell cycle block, but a senescence specific effect, at least in mouse cells.

B23 is a nucleolar phospho-protein, which plays a role in cellular proliferation, cell survival, ribosomal biogenesis, aiding in ribosome assembly [52] and also surveillance of oncogenic insults in cells [53]. In the present study, we found that upon sodium butyrate treatment, the ribosomal biogenesis in the nucleolus is down-regulated with B23 being relocated from the nucleolus to the nucleoplasm of mouse fibroblast. Apart from its proliferative and nucleolar role, several lines of evidence indicate that B23 can be used as a biomarker for hematopoietic leukaemia [54]. Several point mutations on B23 have been detected in Acute Myelogenous Leukemia (AML) patients that also have been shown to translocate B23 protein from nucleolus to the nucleus and cytoplasm. It has also been shown that when cells are stressed with UV irradiation, B23 moves away from the nucleolus [41]. Hence, B23 may play a central role in influencing cellular proliferation and senescence in mammalian cells.

Our study is the latest addition to a growing body of literature on the use of SILAC-based proteomics to examine nucleolar proteome dynamics after metabolic perturbations [30,31,55,56]. These studies build a picture of the divergent responses of the nucleolar composition to different treatments. The change in the nucleolar proteome in senescence reported here is different from each of the published studies, indicating that different treatments are interpreted by mammalian cells differently, and can induce specific biochemical responses in the nucleoli. For example, the redistribution of B23 from the nucleolus, observed in this study and other studies as well [41,57], was not observed in etoposide treatment [53] regardless of the p53 background of the cells studied [56]. This suggests that the B23 re-localization may be a reflection of a certain subset of growth inhibition, including senescence, and it is important to consider the global changes of nuclear proteins, instead of just one or two marker proteins.

## Conclusion

In conclusion, this study describes for the first time the global change in the nucleolar proteome in response to induced senescence in mammalian cells. It provides the foundation on which future work on the role of nucleoli and nuclear organization in senescence can be built. In particular, the combined redistribution of B23 and chromatin in the nucleus found in all three in vitro models of senescence used in this study suggests a possible morphological signature of senescence. The functional significance of this redistribution of nucleolar protein will be a subject of future investigation.

## Abbreviations

B23: (Nucleoplasmin); MTX: (Mitoxantrone); SA-βgal: (Senescence Associated βgalactosidase); 5-FU: (5-Fluorouridine).

## Authors' contributions

BK carried out the experiments and co-wrote the manuscript; BL and ZZ provided Lamin +/+ and Lamin -/- primary mouse cells and also contributed to several scientific discussions related to the project; YWL directed the research and co-wrote the manuscript. All authors read and approved the final manuscript.

## Supplementary Material

Additional file 1 **Isolation of nucleolus in NIH3T3 cells**. (A) Microscopic analysis of an aliquot of the nuclei and nucleolus during the isolation procedure to assess the integrity of the nuclear and nucleolar membrane (B) Western blotting analysis of the nucleolar, nuclear and cytosolic lysate with a specific antibody towards a nucleolar (anti-fibrillarin), nucleoplasmic (anti-Lamin A/C) or cytoplasmic (anti actin) protein marker to test the purity of the cellular fractions. The protein concentration of the lysates were determined using Bradford-Lowry reagent and 10 ug of lyastes from each fraction were run on a SDS-PAGE. Click here for file

Additional file 2 **A list of 344 proteins detected by Quantitative Mass spectrometry showing the SILAC ratio (log 2) of nucleolar proteins in NIH3T3 cells following Sodium Butyrate treatment**. This table lists the accession number with Gene name of 344 proteins identified by Mass spectrometer and generated by using ProteinCenter (Proxeon Bioinformatics), a proteomics data mining and management software which converted protein IDs to gene symbols and perform initial gene ontology characterization. Click here for file

Additional file 3 **Down-regulation of B23 protein level during senescence is not a result of cell cycle block**. (A) NIH3T3 cells were grown either in 10% serum or no serum for 24 hrs. The control or starved cells were stained with propidium iodide to analyze the DNA content by a FACS analysis. (B) The percentage of cells in different phases of the cell cycle with or without serum starvation was analyzed by using ModFit V 3.0 software. (C) NIH3T3 cells were fractionated and pure nuclei from the control and serum starved cells were isolated. The total protein concentration was measured using Bradford-Lowry reagent and equal amounts (10 ug) of nucleoli from the control and serum starved cells were ran on an SDS-PAGE to assess the level of B23 protein during G1 cell cycle block. Click here for file

Additional file 4 **MTX treatment in NIH3T3 cells (A) A FACS analysis of the control, 2 days or 9 days of MTX treated cells stained with propidium iodide**. (B) The percentage of untreated or MTX treated NIH3T3 cells in different phases of the cell cycle analyzed by using ModFit V 3.0 software. (C) Western blotting analysis of the nucleolar, nucleoplasmic and cytosolic lysate from MTX (9 days) treated NIH3T3 cells with a specific antibody towards a nucleolar (anti-B23), nucleoplasmic (anti-Lamin A/C) or cytoplasmic (anti actin) protein marker. The protein concentration of the lysates from the different cellular fraction were determined using Bradford-Lowry reagent and 10 ug of lyastes from each fraction were run on a SDS-PAGE. Click here for file
