# Supplementary figures and images for: Quantitative nucleolar proteomics reveals nuclear re-organization during stress- induced senescence in mouse fibroblast
Source: BMC Cell Biol. 2011 Aug 11;12:33. doi: 10.1186/1471-2121-12-33 (PMC3163619; doi:10.1186/1471-2121-12-33)

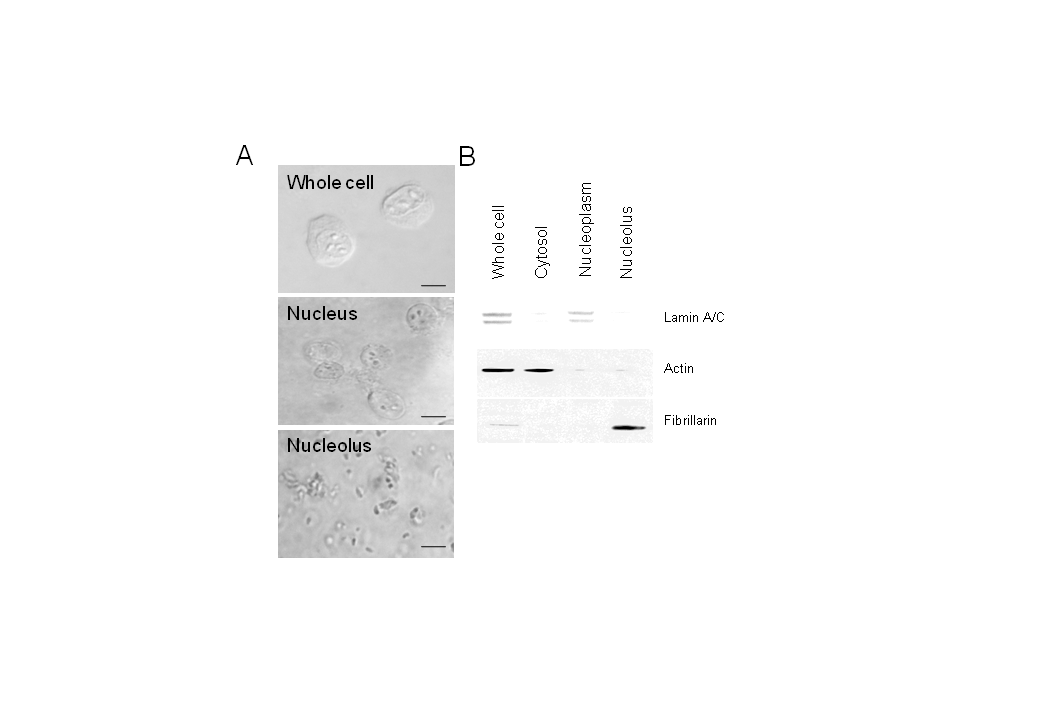

Supplement: Additional file 1 — Isolation of nucleolus in NIH3T3 cells. (A) Microscopic analysis of an aliquot of the nuclei and nucleolus during the isolation procedure to assess the integrity of the nuclear and nucleolar membrane (B) Western blotting analysis of the nucleolar, nuclear and cytosolic lysate with a specific antibody towards a nucleolar (anti-fibrillarin), nucleoplasmic (anti-Lamin A/C) or cytoplasmic (anti actin) protein marker to test the purity of the cellular fractions. The protein concentration of the lysates were determined using Bradford-Lowry reagent and 10 ug of lyastes from each fraction were run on a SDS-PAGE. [file 1471-2121-12-33-S1.TIFF]

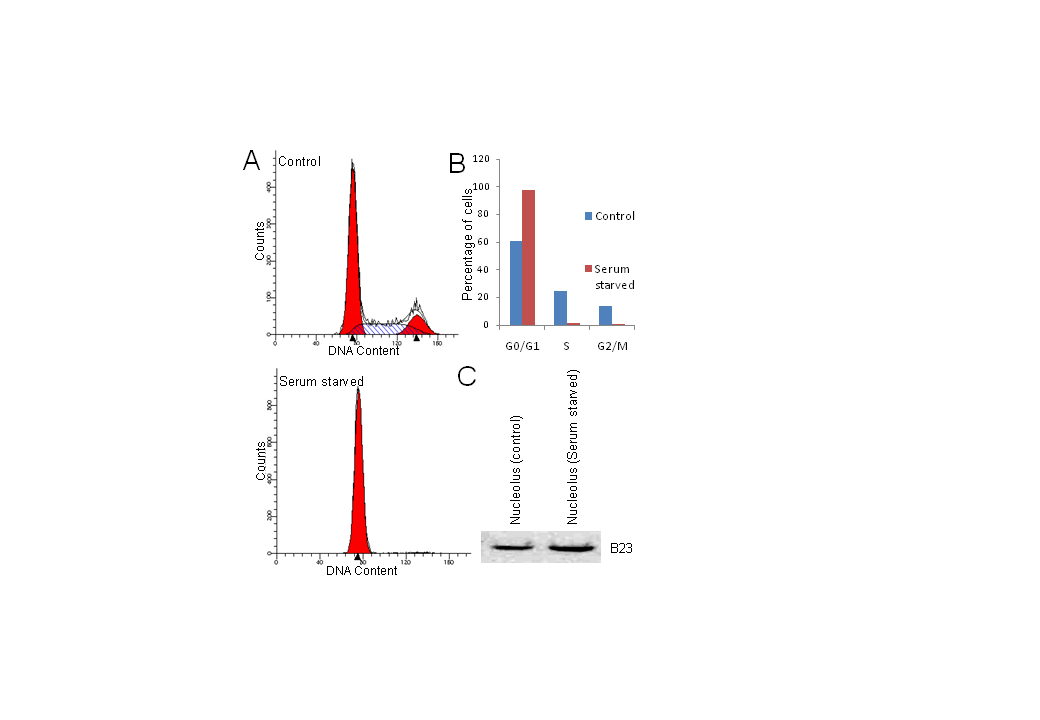

Supplement: Additional file 3 — Down-regulation of B23 protein level during senescence is not a result of cell cycle block. (A) NIH3T3 cells were grown either in 10% serum or no serum for 24 hrs. The control or starved cells were stained with propidium iodide to analyze the DNA content by a FACS analysis. (B) The percentage of cells in different phases of the cell cycle with or without serum starvation was analyzed by using ModFit V 3.0 software. (C) NIH3T3 cells were fractionated and pure nuclei from the control and serum starved cells were isolated. The total protein concentration was measured using Bradford-Lowry reagent and equal amounts (10 ug) of nucleoli from the control and serum starved cells were ran on an SDS-PAGE to assess the level of B23 protein during G1 cell cycle block. [file 1471-2121-12-33-S3.TIFF]

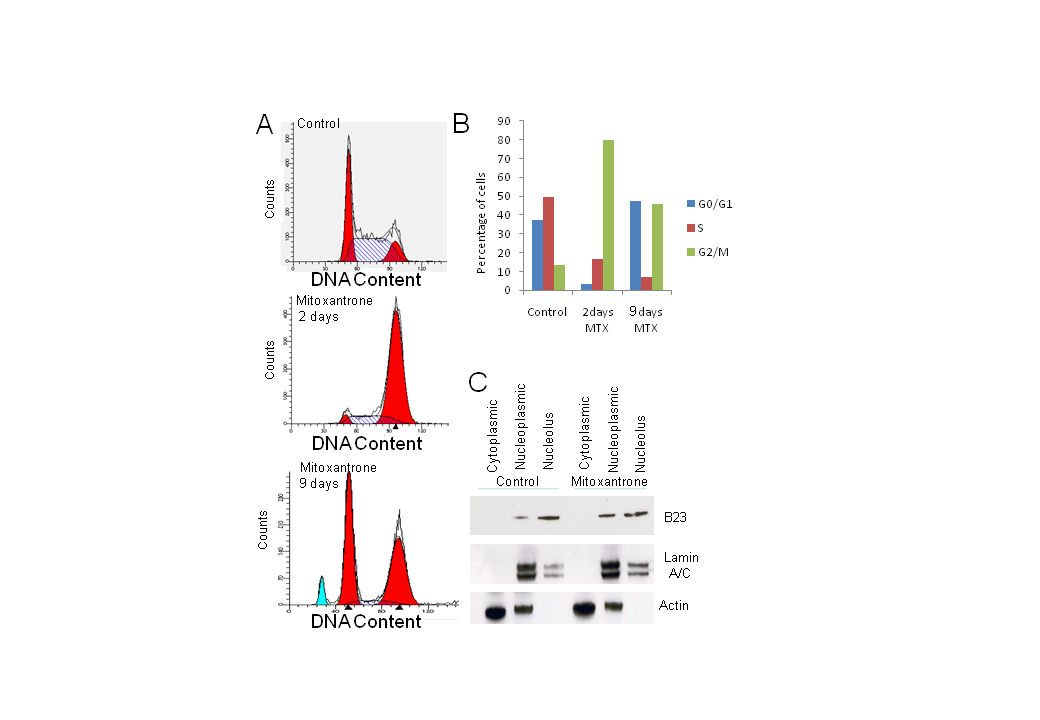

Supplement: Additional file 4 — MTX treatment in NIH3T3 cells (A) A FACS analysis of the control, 2 days or 9 days of MTX treated cells stained with propidium iodide. (B) The percentage of untreated or MTX treated NIH3T3 cells in different phases of the cell cycle analyzed by using ModFit V 3.0 software. (C) Western blotting analysis of the nucleolar, nucleoplasmic and cytosolic lysate from MTX (9 days) treated NIH3T3 cells with a specific antibody towards a nucleolar (anti-B23), nucleoplasmic (anti-Lamin A/C) or cytoplasmic (anti actin) protein marker. The protein concentration of the lysates from the different cellular fraction were determined using Bradford-Lowry reagent and 10 ug of lyastes from each fraction were run on a SDS-PAGE. [file 1471-2121-12-33-S4.TIFF]
